# Mapping of Complete Set of Ribose and Base Modifications of Yeast rRNA by RP-HPLC and Mung Bean Nuclease Assay

**DOI:** 10.1371/journal.pone.0168873

**Published:** 2016-12-29

**Authors:** Jun Yang, Sunny Sharma, Peter Watzinger, Johannes David Hartmann, Peter Kötter, Karl-Dieter Entian

**Affiliations:** Institute of Molecular and Cellular Microbiology Goethe University, Frankfurt am Main, Germany; Scripps Research Institute, UNITED STATES

## Abstract

Ribosomes are large ribonucleoprotein complexes that are fundamental for protein synthesis. Ribosomes are ribozymes because their catalytic functions such as peptidyl transferase and peptidyl-tRNA hydrolysis depend on the rRNA. rRNA is a heterogeneous biopolymer comprising of at least 112 chemically modified residues that are believed to expand its topological potential. In the present study, we established a comprehensive modification profile of *Saccharomyces cerevisiae’s* 18S and 25S rRNA using a high resolution Reversed-Phase High Performance Liquid Chromatography (RP-HPLC). A combination of mung bean nuclease assay, rDNA point mutants and snoRNA deletions allowed us to systematically map all ribose and base modifications on both rRNAs to a single nucleotide resolution. We also calculated approximate molar levels for each modification using their UV (254nm) molar response factors, showing sub-stoichiometric amount of modifications at certain residues. The chemical nature, their precise location and identification of partial modification will facilitate understanding the precise role of these chemical modifications, and provide further evidence for ribosome heterogeneity in eukaryotes.

## Introduction

Ribosomes are large highly conserved ribonucleoprotein complexes that are responsible for protein synthesis [1]. In eukaryotes including *Saccharomyces cerevisiae*, a functional ribosome comprises of two asymmetric subunits, a small 40S and a large 60S [2]. A small subunit (SSU) of the ribosome (40S) in yeast contains a single 18S rRNA of 1800 nucleotides (nts) together with 33 ribosomal proteins [3]. The 60S or the large subunit (LSU) of the yeast ribosome contains three rRNAs: 25S (3396 nts), 5.8S (158 nts) and 5S (121 nts), and 46 ribosomal proteins [3]. The 40S decodes the genetic information carried by mRNA, whereas the 60S catalyzes the joining of amino acids [4–6].

Ribosome biogenesis is a directional process starting in the nucleolus and concluding in the cytoplasm [7]. The complete process of ribosome biogenesis involves more than 200 *trans* acting factors that assist the sequential assembly of ribosomal proteins on rRNA [7]. The majority of the steps involved in the synthesis of ribosomes take place in the nucleolus: a membrane-less and highly dense region within the nucleus built around the rDNA in yeast (*S*. *cerevisiae*) [7].

During the course of ribosome synthesis, sophisticated complexes comprising of both snoRNA guided and “protein-alone” enzymes chemically modify several highly conserved residues in the rRNA of both large and small subunit of the ribosomes [8,9]. Ribosomal RNA contains three types of chemical modifications, 2′-O methylation of ribose sugars (Nm), base isomerization (pseudouridylations (Ψ)), and base modifications (methylations (mN) and acetylations (acN)) [10]. Methylated ribose sugars and pseudouridines represent the majority of rRNA modifications [11]. A methylated sugar is generated by the addition of one methyl group at the 2′-OH position of the ribose on the nucleoside and is independent of the nature of the base. Pseudouridylation results from uridine isomerization, involving a 180° rotation around the N3–C6 axis [12].

Box C/D snoRNPs (small nucleolar ribonucleoproteins) catalyze site-directed methylation at the 2′-OH position on the sugar of the targeted nucleotide, whereas the box H/ACA snoRNPs isomerize targeted uridine to pseudouridine [13,14]. Remarkably, the substrate specificity for both ribose methylations and pseudouridylations is dictated by the RNA component of these snoRNP enzymes. Here the complementary sequences in the guide RNA base pair with target RNAs to decide the nucleotide for modification [13,14]. The catalytic activities are provided by the methyltransferase (Nop1 or Fibrillarin) or the pseudouridine synthase (Cbf5), respectively [15,16]. In contrast, base modifications are catalyzed by “protein only” enzymes [10].

The current information on the location and chemical nature of these chemical modifications, especially ribose methylations in yeast date back to “70s”, and have been generated by the use of TLC (**T**hin **L**ayer **C**hromatography) and primer extension [17]. Although these techniques have been successful in providing the preliminary information, they still lack the sensitivity and quantitative information as compared with the state of art technologies like RP-HPLC and mass spectrometry [18–20].

To analyze and explore the significance of these modifications in ribosome biogenesis and ribosome function, a comprehensive analysis of their chemical nature and precise location on the ribosome is central.

In the present study using Mung Bean Nuclease (MBN) protection assay, snoRNA deletion mutants and rDNA mutants, we systematically mapped the ribose methylations and base modifications of both small and large subunit rRNA and provide further evidences for eukaryotic ribosome heterogeneity.

## Materials and Methods

### Yeast strains and media

All yeast strains were grown at 30°C in YPD medium (1% of yeast extract, 2% of peptone, 2% of glucose) or in synthetic dropout medium (0.5% of ammonium sulphate, 0.17% of yeast nitrogen base, 2% of glucose). The yeast strains used in the present study are listed in S1 Table.

### Extraction of total RNA and isolation of intact 18S rRNA and 25S rRNA

**Fig 1:**
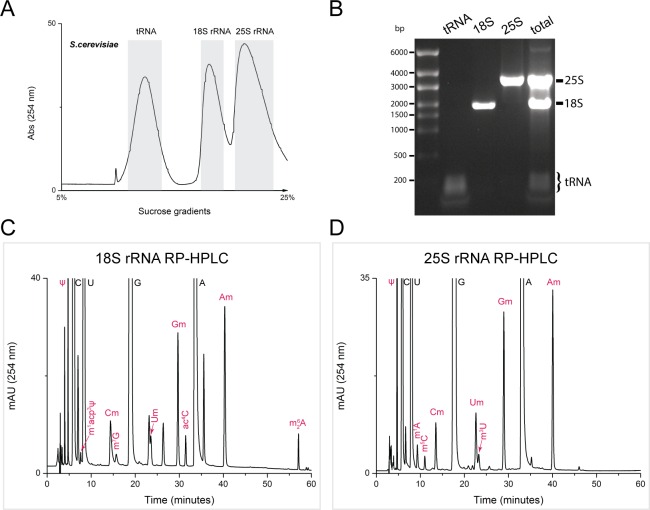
Reversed Phase High Performance Liquid Chromatography (RP-HPLC) analysis of 18S and 25S rRNA of S. cerevisiae (budding yeast). 18S and 25S rRNA of yeast were isolated by sucrose gradient centrifugation. A) Sucrose gradient sedimentation profile of yeast total RNA separated on 5% to 25% sucrose gradient. Fractions corresponding to tRNA (predominantly), 18S rRNA, and 25S rRNA were collected, and the rRNAs were isolated using 95% ethanol precipitation at -80°C. B) 1.5% Agarose gel showing the rRNA recovered after ethanol precipitation. Yeast total RNA was used as a control. Both 18S and 25S rRNA were digested to nucleosides using P1 nuclease and alkaline phosphatase. Nucleosides derived from 18S rRNA and 25S rRNA were analyzed by RP-HPLC. C) RP-HPLC chromatogram of 18S rRNA, and D) 25S rRNA, showing peaks corresponding to major nucleosides labeled in black and of modified residues in red.

Total RNA from yeast was isolated using hot phenol method as described by Collart *et al*. [21]. For isolation of yeast 18S and 25S rRNA, 500 μg of total RNA was layered onto a 5–25% sucrose gradient in TEN buffer (100 mM Tris-HCl pH 7.8, 100 mM NaCl and 1mM EDTA in nuclease free water). The gradient was made with Gradient Master 107 (Biocomp). Samples were then centrifuged at 23,000 rpm for 25 hours (h) at 4°C in an SW40 rotor in an L- 70 Beckman ultracentrifuge. The gradients were fractionated in an ISCO density gradient fractionator. Fractions corresponding to 18S and 25S rRNA were collected and intact 18S and 25S rRNA were extracted by overnight ethanol precipitation. The integrity of RNA was analyzed using 1.5% agarose gel stained with ethidium bromide (Fig 1B).

### Mung bean nuclease protection assay

Mung bean nuclease (MBN) protection assay was used exactly as described before [22]. 1000 pmol of the synthetic deoxy oligonucleotides were incubated with 100 pmol of rRNA (18S or 25S) and 5% of DMSO in 0.3 volume of hybridization buffer (250 mM of HEPES, 500 mM of KCl at pH 7). The mixture was incubated at 90°C for 5 minutes and then slowly cooled down to 45°C over 2 h. After hybridization, 35 units of mung bean nuclease (New England Bio Labs (NEB)) and 0.02 mg/ml RNase A (Sigma—Aldrich) along with appropriate amount of 10X MBN buffer (NEB) were added to start digestion. The digestion was carried out at 35°C for 1 h. After digestion, the protected fragment (RNA-DNA hybrid) was extracted from the reaction mixture by phenol/chloroform extraction, followed by overnight ethanol precipitation. The protected rRNA fragments were next separated from the complementary DNA oligonucleotides on a denaturing 7 M Urea 13% PAGE gel. Bands were visualized by ethidium bromide staining and the rRNA band was excised and eluted using the D-Tube TM Dialyzers according to the manufacturer’s protocol for electro elution (Novagen). All synthetic oligonucleotides used in the present study are listed in S2 Table.

### Reversed Phase High Performance Liquid Chromatography

Nucleosides for RP-HPLC were prepared as described by Gehrke and Kuo [18] and adapted for rRNA as described previously [23]. For the preparation of nucleosides, only purified and intact 18S (100 pmoles) and 25S (60 pmoles) rRNA were used. The rRNA (18S or 25S rRNA) was denatured for 2 minutes on a heating block in an eppendorf cup at 100°C, followed by rapid cooling on ice. Five microliter of 10 mM ZnS0_4_, 10 μl P1 nuclease (Sigma) (200 units per ml in 30 mM sodium acetate, pH 5.4) were next added to the cup. Nuclease digestion was carried out at 37°C. After 16 h, 10 μl of 0.5 M Tris buffer, pH 8.3, and 10 μl bacterial alkaline phosphatase (Sigma) (100 units per ml in 2.5 M ammonium sulphate) were added to the cup and incubated at 37°C for 2 h. The nucleosides hydrolysates were clarified by centrifugation at 13 000 rpm.

Nucleosides were analyzed by RP- HPLC on a Supelcosil LC-18-S HPLC column (25 cm x 4.6 mm, 5 mm) equipped with a pre-column (4.6 × 20 mm) at 30°C on an Agilent 1200 HPLC system, using a gradient elution described previously in [18]. For m^3^U a different elution protocol described elsewhere was used [24]. In contrast to gradient elution for rest of the modified nucleosides, the elution conditions for m^3^U were changed to an isocratic mode using 50% buffer A (10 mM of NH_4_H_2_PO_4_, 2.5% of methanol at pH 5.3) and 50% buffer B (10 mM of NH_4_H_2_PO_4_, 20% of methanol at pH 5.1).

### Quantification of the modified nucleosides molar ratios

Approximate modification levels for each ribose and base modifications were calculated from HPLC peak areas, by dividing the total peak area by their standard molar response factors as established previously by Gehrke and Kuo [18] and by Noon *et al*. [25,26]. Mole % (Seq) was calculated assuming that 18S rRNA (1800 nts) contains 473 unmodified adenosines (A), 494 unmodified uridines (U), 453 unmodified guanosines (G), and 343 unmodified cytidines (C), and similarly for 25S rRNA (3396 nts) assuming that it contains 885 unmodified adenosines (A), 828 unmodified uridines (U), 956 unmodified guanosines (G), and 653 unmodified cytidines (C). Likewise, for MBN protected fragments residues per moles or extent of modifications for each residue were established assuming either 50 nts (18S rRNA) or 60 nts (25S rRNA) and its comparison to the residues/moles calculated from the 18S rRNA. For example, since residue per mole for 2’-O-Methylcytidines (Cms) from an intact 18S rRNA were calculated from HPLC peak area to be 3.01 close to their actual value of 3.0, this provided initial indications that these residues are fully modified.

## Results

### High resolution composition analysis of 18S rRNA and 25S rRNA by RP-HPLC

To analyze the composition of 18S rRNA and 25S rRNA by high resolution RP-HPLC, the retention time and molar response factors (at 254 nm) of major and modified nucleosides were first established on our HPLC system. Commercially available purified nucleosides from CarboSynth Ltd., Berkshire (UK) were used as references (S1 Fig). Interestingly, values for both the retention time and the response factors obtained on our system were similar to the values calculated previously by Gehrke and Kuo [18]. We also assessed our method for linearity by calibration point standard curves for all modified nucleosides to avoid deviations from Beer-Lambert´s law.

Intact 18S and 25S rRNA from exponentially growing yeast culture were isolated by sucrose gradient density centrifugation (Fig 1A and 1B), and were digested to nucleosides using P1 nuclease and bacterial alkaline phosphatase as explained in Materials and Methods. The composition of the nucleosides derived from both 18S and 25S rRNA were next analyzed by RP-HPLC.

As shown in Fig 1C and 1D, all modified ribonucleosides of both 18S and 25S rRNA could be successfully resolved. The molar amount of all nucleosides were next quantitated using their molar response factors. This permitted accurate quantification of mole percent (%) for each major nucleoside and established approximate number of residues per mole for each modification in 18S or 25S rRNA (Table 1). To ignore any experimental or technical error, the mean values calculated from nine independent runs for each 18S and 25S rRNA are presented.

**Table 1:** RP-HPLC quantification of Nucleosides in S.cerevisiae 18S and 25S rRNA.

	18S rRNA		25S rRNA	
*Nucleosides*	*Mole % (Seq)*	*Mole % (HPLC)*	*Mole % (Seq)*	*Mole %(HPLC)*
**C (Cytidine)**	19.1	19.5 ± 0.48	19.2	19.4 ± 0.56
**U (Uridine)**	27.4	27.1 ± 0.30	24.4	24.9 ± 0.39
**G (Guanosine)**	25.2	25.7 ± 0.12	28.2	27.5 ± 0.17
**A (Adenosine)**	26.3	26.6 ± 0.68	26.1	26.8 ± 0.64
**Residues/mole**				
**m**^**1**^**A (N1-Methyladenosine)**	0	0	2	1.98 ± 0.10
**m**^**5**^**C (C5-Methylcytidine)**	0	0	2	1.81 ± 0.17
**Cm (2’-O-Methylcytidine)**	3	3.1 ± 0.26	7	6.74 ± 0.31
**m**^**7**^**G (N7-Methylguanosine)**	1	0.72*± 0.16	0	0
**m**^**5**^**U (C5-Methyluridine)**	0	0	0	0
**Um (2’-O-Methyluridine)**	2	1.89 ± 0.33	8	7.1 ± 0.31
**m**^**3**^**U (N3-Methyluridine)**	0	0	2	2.1 ± 0.17
**Gm (2’-O-Methylguanosine)**	5	4.91 ± 0.27	10	9.3 ± 0.39
**ac**^**4**^**C (N4-Acetylcytidine)**	2	2.12 ± 0.11	0	0
**Am (2’-O-Methyladenosine)**	8	7.33 ± 0.43	12	11.8 ± 0.48
**m**^**6**^_**2**_**A (N6,N6-Dimethyladenosine)**	2	nc	0	0

Mole % (Seq) was calculated from the number of each nucleoside in 18S and 25S rRNA sequences and as explained in Materials and Methods. Mole % (HPLC) and standard deviation values were calculated from the peak area of the respective nucleosides using their standard response factors, from nine independent runs for each 18S and 25S rRNA. nc–not calculated

* m^7^G has been shown to undergo partial-degradation during hydrolysis [18].

### Systematic analysis of modified residues by mung bean nuclease protection assay and RP-HPLC

Structural and biochemical analyses of ribosomes have provided detailed information about the functional centers of the ribosomes. The exact location of the modified residues on rRNA offers first crucial hints for their likely role in ribosome function.

**Fig 2:**
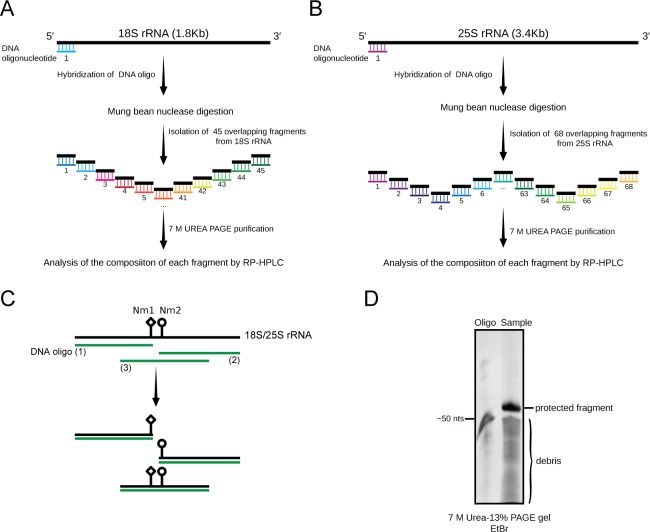
Mung bean nuclease protection assay. A) Schematic illustration of MBN protection assay used in the present study for the analysis of chemical modifications of 18S rRNA, and B) 25S rRNA. 45 distinct fragments from 18S, and 68 separate fragments from 25S rRNA were isolated for mapping of their chemical constituents. C) Graphical illustration for the use of tiling set of overlapping fragments used in the present study to map the modified residues to a single nucleotide resolution. The fragments protected from MBN were isolated from the debris by 7 M Urea 13% PAGE gel. D) Representative gel for the MBN assay showing an intact protected fragment retrieved after the MBN digestion. The synthetic antisense oligonucleotide used for the protection is used as a marker (50 nts). All fragments isolated from 18S and 25S rRNA were extracted from the gel and digested to nucleosides for their composition analysis.

To map the precise location of modified residues in both 18S and 25S rRNA, MBN protection assay was used. Hybridization with suitable oligonucleotides protect the respective area of the rRNA against MBN digestion. This allows isolation of specific fragments from single stranded RNA. Fig 2A and 2B illustrate the mapping strategy used in the present study for isolating different fragments of 18S and 25S rRNA.

Using synthetic antisense oligonucleotides, 45 distinct, overlapping fragments of 50 nucleotides (nts) in size, spanning the whole 18S rRNA (1800 nts), and 68 distinct fragments of 60 nts from the 25S rRNA (3396 nts) were isolated (Fig 2A and 2B). To provide a single nucleotide resolution for each modified residue, the fragments containing modified residues were further scanned by several tiling set of overlapping fragments as exemplified in Fig 2C. We also used rDNA and snoRNA point mutations to validate the location of some of the modified residues (discussed below). After the MBN digestion, all protected fragments were purified over 7 M Urea 13% PAGE gels. These fragments were then digested to nucleosides (as explained above) and their composition were analyzed by RP-HPLC (Fig 2D).

### Mapping of complete set of 18S rRNA ribose methylations and base modifications to a single nucleotide resolution

**Fig 3:**
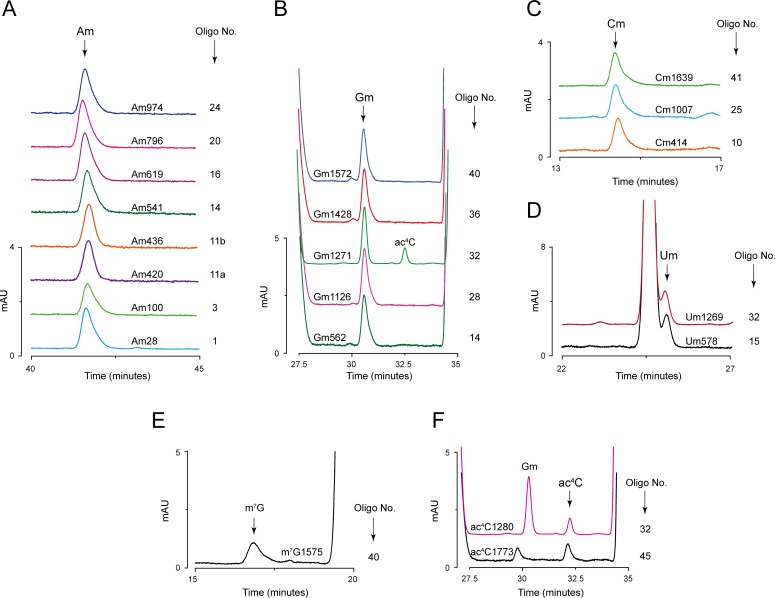
Identification and mapping of complete set of ribose and base modifications of 18S rRNA. Analysis of the composition of 45 fragments derived from 18S rRNA using MBN assay permitted identification of all ribose and base modifications of 18S rRNA. A) Overlaid chromatograms of different fragments identified to contain Am residues; (B) Gm residues; (C) Cm residues; (D) Um residues; (E) m^7^G, and (F) ac^4^C residues. Location of each modification along with the oligonucleotide number used for the isolation of respective fragment are mentioned on the right side of the peak.

**Fig 4:**
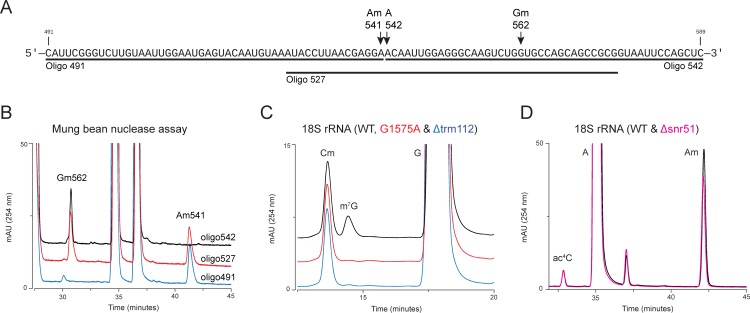
Specific mapping of the chemical modifications of 18S rRNA. To map the chemical modifications with a single nucleotide resolution, tiling set of overlapping fragments, along with snoRNA, and rDNA point mutants were used. To exemplify the tiling set strategy, Am541 mapping used in the present study is shown. A) To map Am 541 to its precise location, three fragments protected by oligo 491, 527, and 542 were isolated. B) Overlaid chromatograms of these three fragments. To validate the precise location of m^7^G1575, rDNA point mutant was generated where G1575 was exchanged with A in a plasmid-borne copy of 35S rDNA transcribed under the native promoter in a strain where the genomic rDNA was deleted. Exchange of G1575 to A led to complete loss of m^7^G derived from 18S rRNA. As a control, we also used ∆trm112 mutant. Loss of trm112 leads to a complete loss of m^7^G1575 [30]. C) Overlaid chromatograms of isogenic Wild type (WT), G1575A rDNA point, and ∆trm112 mutant. To validate partial modifications at Am100 of 18S rRNA, corresponding snoRNA snR51 was deleted and its contribution to the total Am peak of 18S rRNA was assessed. D) Overlaid chromatograms of isogenic WT and ∆snr51.

The composition analyses of all 45 fragments derived from 18S rRNA by RP-HPLC allowed identification of all 18 ribose methylations (Nm) including the recently identified Gm562 [27], and apart from m^1^acp^3^Ψ (N1-methyl-N3-aminocarboxypropyl pseudouridine) [28], identification of all 5 base modifications (methylations and acetylations) of 18S rRNA (Fig 3). Isolation of tiling set of overlapping fragments from the region of 18S rRNA found to be positive for a modification, allowed precise mapping of these modifications to a single nucleotide resolution. As an example, we present mapping of Am541 to its precise location in Fig 4A and 4B.

Since the machinery and the mechanism associated with ribose methylations are very well characterized: the guide sequences direct ribose methylation to the nucleotide base paired to the 5th nucleotide up-stream of the box D or D′ sequence (box D+5 rule) [13], to further validate the precise location of ribose methylated nucleotides, deletion mutants of C/D box snoRNAs (snr40, snR41, snr56, snr57, snr70, and snr79) were used (S2 Fig). Fragments encompassing ribose methylated residues of the 18S rRNA, performed by these snoRNAs were isolated from WT and respective snoRNA deletion mutant, using MBN assay and were subjected to RP-HPLC analysis. A specific loss of peak corresponding to the respective modification in a deletion mutant validated the precise location of the modified residue (Figs 4B and S2). Similarly, we used rDNA point mutant (G1575A) to further validate the presence of m^7^G at position 1575 in the 18S rRNA (Fig 4C).

Since intact rRNA fragments (50 nts) of 18S rRNA with a known amount and sequence (number and chemical nature of nucleotides) were used for the analysis of the composition, we also quantified relative amount of each modification using both molar response factor and relative response factor (calculated using response factors of major nucleosides derived from the same fragment). Mole per cent and residues per mole calculated from analysis of fragments permitted more accurate estimation of extent of modification (Table 2). Based on previous reports of hypomodification and to exclude any experimental error, we considered a threshold of 75% for sub-stoichiometric modifications [29].

**Table 2:** Approximate values for the extent of modifications in 18S and 25S rRNA, using RP-HPLC and its comparison with RiboMethSeq and SILNAS analyses.

Modified residue	Made by	% of modification(RP-HPLC)	% of modification (RiboMethSeq) (Ref.35)	% of modification (RiboMethSeq) (Ref.34)	% of modification (SILNAS) (Ref.36)
**18S rRNA**					
**Am28**	snR74	90% ± 3.8%	85%	99%	>95%
**Am100**	snR51	73% ± 2.4%	77%	77%	80%
**Cm414**	U14	89% ± 3.1%	89%	96%	>95%
**Am420**	snR52	98% ± 1.5%	83%	90%	>95%
**Am436**	snR87	98% ± 1.8%	74%	89%	73%
**Am541**	snR41	95% ± 3.2%	87%	99%	>95%
**Gm562**	snrR40	100%	94%	92%	67%
**Um578**	snR77	95% ± 3.3%	90%	93%	>95%
**Am619**	snR47	98% ± 1.2%	85%	92%	100%
**Am796**	snR53	98% ± 1.1%	92%	97%	>95%
**Am974**	snR54	95% ± 3.4%	91%	96%	94%
**Cm1007**	snR79	100%	92%	98%	>95%
**Gm1126**	snR41	100%	94%	93%	89%
**m^1^acp^3^Ψ1191**	Tsr3	nd	nd	nd	100%
**Um1269**	snR55	95% ± 2.1%	88%	92%	>95%
**Gm1271**	snR40	100%	92%	98%	>95%
**ac^4^C1280**	Kre33	96% ± 1.9%	nd	nd	84%
**Gm1428**	snR56	100%	95%	99%	>95%
**Gm1572**	snR57	100%	89%	97%	100%
**m^7^G1575**	Bud23	72*% ± 2.8%	nd	nd	>95%
**Cm1639**	snR70	92% ± 2.9%	93%	98%	79%
**ac^4^C1773**	Kre33	100%	nd	nd	>95%
**m^6^_2_A1781**	Dim1	nc	nd	nd	90%
**m^6^_2_A1782**	Dim1	nc	nd	nd	90%
**25S rRNA**					
**m^1^A645**	Rrp8	99% ± 1%	nd	nd	>95%
**Am649**	U18	93% ± 2.5%	88%	98%	>95%
**Cm650**	U18	100%	95%	98%	94%
**Cm663**	snR58	81% ± 4.3%	62%	91%	74%
**Gm805**	snR39b	90% ± 4.7%	93%	98%	>95%
**Am807**	snR39_snR59	98% ± 1%	94%	97%	>95%
**Am817**	snR60	98% ± 0.8%	92%	85%	89%
**Gm867**	snR50	90% ± 3.7%	95%	95%	78%
**Am876**	snR72	95% ± 2.2%	89%	91%	75%
**Um898**	snR40	94% ± 1.8%	78%	85%	94%
**Gm908**	snR60	95% ± 1.1%	96%	96%	100%
**Am1133**	snR61	98% ± 0.5%	92%	99%	88%
**Gm1142**	?(snr57)	absent	absent	absent	absent
**Cm1437**	U24	100%	92%	97%	>95%
**Am1449**	U24	97% ± 0.7%	67%	99%	>95%
**Gm1450**	U24	100%	94%	99%	>95%
**Um1888**	snR62	99% ± 1.3%	94%	97%	>95%
**m^1^A2142**	Bmt2	99% ± 0.7%	nd	nd	>95%
**Cm2197**	snR76	100%	71%	86%	91%
**Am2220**	snR47	98% ± 3.1%	94%	99%	93%
**Am2256**	snR63	98% ± 2.3%	94%	95%	100%
**m^5^C2278**	Rcm1	100%	nd	nd	100%
**Am2280**	snR13	99% ± 0.6%	91%	100%	100%
**Am2281**	snR13	99% ± 0.9%	92%	100%	100%
**Gm2288**	snR75	100%	95%	96%	>95%
**Cm2337**	snR64	100%	94%	99%	100%
**Um/Ψm2347**	snR65/snR9	66% ± 3.7%	69%	61%	76.2%/12.9%
**Gm2395**	?snR190	absent	absent	absent	absent
**Um2417**	snR66	99% ± 0.1%	95%	99%	100%
**Um2421**	snR78	99% ± 1.5%	86%	93%	>95%
**Gm2619**	snR67	100%	93%	98%	87%
**m^3^U2634**	Bmt5	100%	nd	nd	>95%
**Am2640**	snR68	95% ± 3.6%	90%	96%	93%
**Um2724**	snR67	92% ± 2.1%	92%	98%	>95%
**Um2729**	snR51	89% ± 4.3%	67%	94%	75%
**Gm2791**	snR48	95% ± 2.3%	94%	98%	86%
**Gm2793**	snR48	95% ± 1.9%	95%	98%	>95%
**Gm2815**	snR38	95% ± 2%	95%	99%	>95%
**m^3^U2843**	Bmt6	100%	nd	nd	>95%
**m^5^C2870**	Nop2	100%	nd	nd	>95%
**Um2921**	snR52	93% ± 2.2%	92%	99%	>95%
**Gm2922**	Spb1	97% ± 3.5%	95%	98%	>95%
**Am2946**	snR71	99% ± 0.6%	92%	99%	91%
**Cm2948**	snR69	99% ± 0.8%	77%	82%	>95
**Cm2959**	snR73	93% ± 4.1%	96%	95%	94%

nd–not detected; nc–not calculated.? (snRx)- predicted according to the guide sequence; lacks experimental evidence. % of modification was calculated as a mean of three independent calculations ± Relative standard deviations (RSD).

* m^7^G has been shown to undergo partial-degradation during hydrolysis [18].

As shown before by DNAzymes and mass spectrometry, we also observed partial modification at Am100 by RP-HPLC [29]. For further validation of the amount of modified nucleosides, single deletion mutants of snoRNAs involved in modifying identical nucleoside at distinct positions were generated and a reduction in the amount of total peak area was calculated. As calculated from the chromatograms shown in Fig 4D, peak area in *∆snr51* mutant (7 Am residues) and its comparison with peak area of Am residues in the WT (8 Am residues) further supported a partial modification at Am100 (approximately 73%). Similarly, calculation of all Gm and Cm modifications in the 18S rRNA revealed that these residues are relatively fully methylated (S2 Fig).

As far as base modifications are concerned, both acetylated cytidines are nearly fully modified at their respective locations. Quantification of the extent of modification was also validated by analyzing the rDNA point mutant (C1773G), where exchange of C1773G led to nearly 50% reduction in the total amount of ac^4^Cs from the total 18S rRNA (S2G Fig). Intriguingly, extent of modification at m^7^G 1575 was calculated to be 72%, suggesting a hypomethylation at 1575 (Tables 1 and 2). However, since previous studies have highlighted partial loss of m^7^G during hydrolysis [18], at present we are not able to rule out this to be the cause for hypomodification at m^7^G 1575. Relatedly, unavailability of commercial reference for m^6^_2_A precluded us from calculating the extent of modifications at 1781 and 1782.

### Mapping of complete set of 25S rRNA ribose methylations and base modifications to a single nucleotide resolution

**Fig 5:**
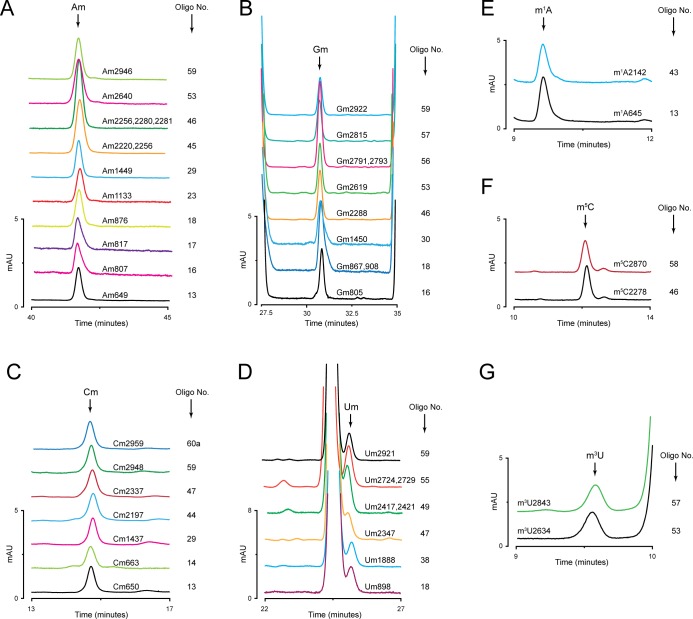
Identification and mapping of complete set of ribose and base modifications of 25S rRNA. Composition analysis of the 68 discrete fragments isolated from 25S rRNA using MBN protection assay. Overlaid RP-HPLC chromatograms of different fragments identified to contain (A) Am residues; (B) Gm residues; (C) Cm residues; (D) Um residues; (E) m^1^A; (F) m^5^C; and (G) m^3^U residues. Location of each modification along with the oligonucleotide number used for the isolation of respective fragment are stated on the right side of the peak. For the fragments containing more than one modifications, e.g. oligo 45, oligo 46 for Am, oligo 18 and 56 for Gm, and oligo 49 and 55 for Um, strategy explained above in Fig 2C were used to map them to exact position.

**Fig 6:**
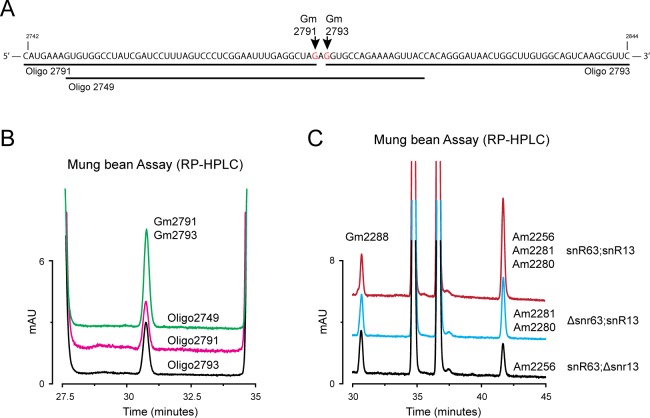
Precise mapping of the chemical modifications of 25S rRNA. For the mapping of chemically identical modifications located adjacent to each other on 25S rRNA, tiling set of overlapping fragments, along with snoRNA deletion mutants were used. The mapping strategy for Gm2791 and Gm2793 to their precise location used in the present study is shown here as an example. A) To map Gm2791 and Gm2793 to their precise location, three fragments protected by oligo 2749, 2791, and 2793 were isolated. B) Overlaid chromatograms of these three fragments. Similarly, to validate the mapping of Am2256, Am2289 and Am2281, we used respective snoRNA deletion mutant–snR63 for Am2256, and snR13 for both Am2280 and Am2281. Where loss of snR13 led to approximately two third reduction in Am peak area, loss of snR63 resulted in only one third reduction. Gm2288 peak remained unaltered and was used as an internal control for the quantification analysis.

Like 18S rRNA, analysis of the composition of 68 fragments of 60 nts each derived from 25S rRNA identified all 37 ribose methylations and 6 base methylations– 12 Ams, 10 Gms, 8 Ums, and 7 Cms, along with 2 m^1^A, 2 m^3^U and 2 m^5^C (Fig 5). A similar strategy of tiling set of overlapping fragments as used for 18S rRNA were used to map these residues to the single nucleotide resolution. As shown in Fig 6, we mapped Gm2791 and Gm2793 to their respective locations. Besides overlapping fragments, snoRNA deletion mutants (Fig 6C) and rDNA point mutants for all base methylations (except m^5^C2870) were also used (S3 Fig). All modified residues with their precise locations are listed in Table 2.

All ribose and base methylations of 25S rRNA were also quantified. According to our calculations based on the molar response factor, apart from partial modification at Cm663 and Um2347 all other residues were found to be relatively fully modified (Table 2).

We could not extend our current protocol to the mapping of pseudouridylations (Ψ) due to overlapping retention time for Ψ and contaminants (either deoxy cytidine (dC) or deoxy uridine (dU)) from the synthetic oligonucleotides used for MBN digestion.

### Mapping of the modified nucleosides on 2D and 3D structures of yeast ribosomal rRNA

**Fig 7:**
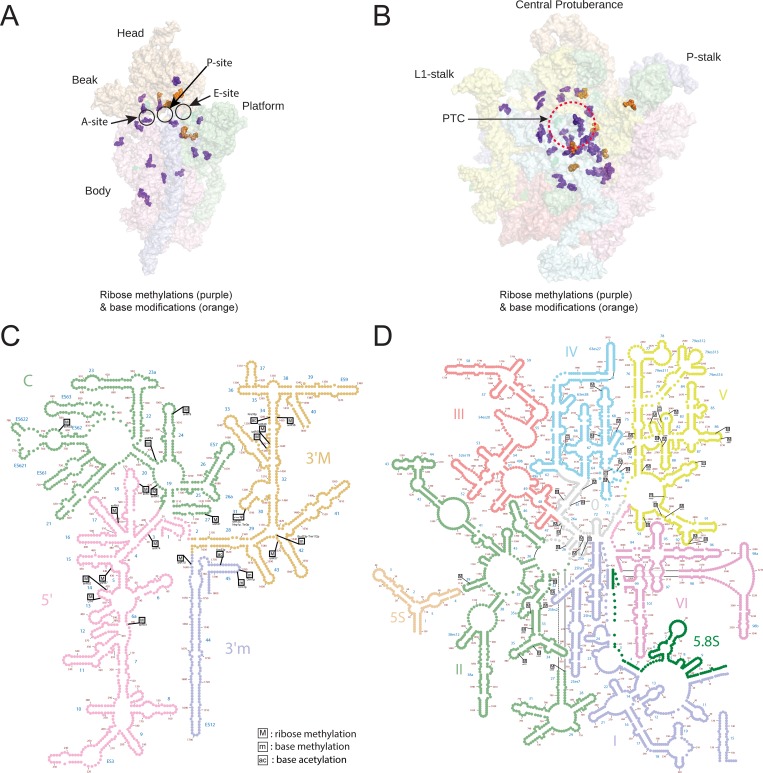
3D and 2D modification atlas for the chemical modifications of yeast rRNA. Both ribose and base modifications analyzed in the present study are mapped on the 3D structure of ribosome. 3D cartoon of the yeast 18S rRNA (A) and 25S rRNA (B), highlighting the location of ribose methylations (purple spheres), and base modifications (orange spheres). PDB files 3U5B and 3U5D were used for the representation of 18S and 25S ribosomal RNA. The cartoon was made by PyMOL software (PyMOL Molecular Graphics System, Version 1.2r3pre, Schrödinger, LLC.). Both ribose and base modifications were also mapped on to the 2D sequence map of the 18S (C) and 25S (D) rRNA of the yeast using online RiboVision suite (http://apollo.chemistry.gatech.edu/RiboVision/).

Once the chemical modification profile of 18S and 25S rRNA along with their precise location on the primary structure was established, we next mapped ribose and base modifications on both 2D and 3D structures of both yeast 18S and 25S rRNA. As shown in Fig 7, majority of modified residues are located proximal to the functional centers of the ribosomes–decoding center (equivalent of A site of 40S), peptidyl transferase center (A and P site of 60S). Another interesting location of many of these chemical modifications are in the intersubunit region–this region is responsible for keeping two subunits of ribosome together by a series of bridges, involving RNA–RNA, RNA–protein and protein–protein interactions. Previous work has shown that the loss of these modifications in the intersubunit regions influence both ribosome structure and function [31]. Interestingly, more similar scenarios for the possible role of these modifications in the intersubunit region has been recently discussed, where a eukaryotic specific bridge eB14 formerly shown to be formed by ribosomal protein eL41 and rRNA [32] is found to be apparently formed by interaction of eL41 with three of the base modifications on the small subunit (ac^4^C1773 and two dimethylations m^6^_2_A1781 and 1782) and m^5^C2278 on the large subunit rRNA [10]. This further accentuates an important function of these modifications not only in the catalytic core but also around the intersubunit region of the ribosomes. Moreover, since intersubunit interactions are also crucial for late processing of small subunit, these modifications might also act as quality control checkpoints during ribosome biogenesis [10,33].

## Discussion

In the present study, we subjected rRNA of *S*. *cerevisiae* to high resolution composition analysis by RP-HPLC and mapped all ribose and base methylations of both 18S and 25S rRNAs to a single nucleotide resolution. Our analysis revealed that as predicted by Rudi Planta´s lab and documented recently by RiboMethSeq, *S*. *cerevisiae* indeed contains total of 55 ribose methylations– 18 on the 18S rRNA and 37 on the 25S rRNA [17,34,35]. Our RP-HPLC analysis also invalidated the presence of Gm1142 and Gm2395 in 25S rRNA [13,36]. Although we cannot exclude the presence of any additional modification that results in a different retention time, which might have precluded its identification by our RP-HPLC analysis. We mapped all six base methylations– 2 m^1^A, 2 m^5^C and 2 m^3^U of the 25S rRNA to a single nucleotide resolution and invalidated presence of any m^5^U residues in the 25S rRNA [37]. Recent mass spectrometry analysis by Taoka *et al*. has also invalidated the existence of m^5^U and Gm1142 and Gm2395 in the 25S rRNA [35–37]. This study also identified Ψm at 2347 [36], which we could not confirm as we could not detect a Ψm peak on our chromatograms.

As discussed before, since the precise locations of the modifications are absolutely crucial, we would like to point out the discrepancies between our findings and the previous numbering of the modified residues in 25S rRNA by Taoka *et al*. [36], where all residues are shifted by minus 2 nts in numbering, e.g. the correct position for m^1^A residues are 645 and 2142, instead of 643 and 2140.

Reversed Phase-High Performance Liquid Chromatography (RP-HPLC) with UV detection is a powerful and robust tool for both quantitative and qualitative analysis of nucleosides. RP-HPLC not only allows easier separation of canonical nucleosides of RNA like A, U, G and C, but also of a large number of modified nucleosides, based on their interaction with the polar mobile phase and nonpolar stationary phase. Reproducible retention time and high molar absorptions of nucleosides provide RP-HPLC both a qualitative and quantitative edge.

RP-HPLC provides a relatively economical yet powerful and high resolution chromatographic analysis of modified nucleosides [18]. Furthermore, commercial availability of nearly all modified nucleosides make HPLC analysis even more robust and economical in terms of identification and quantification of the modified residues. Nevertheless, it is important to mention that for modified nucleosides with similar or uncharacterized retention time, HPLC coupled with only UV-detector is limited for both qualitative and quantitative analysis. This limitation can be resolved either by collecting and analyzing the eluate by mass spectrometry or by directly coupling the HPLC to the mass spectrometer (HPLC-MS or LC-MS). Herein, utilizing standard molar response factors, we calculated approximate magnitude of modifications level for ribose and base modifications, and validated it with parallel analysis of both snoRNA and rDNA mutants for a number of residues. Parallel analysis of the mutants allowed us to reduce the uncertainty levels arising due to possible technical errors. Although the estimated values for extent of modification obtained by RP-HPLC differ at certain residues from both SILNAS [36] and RiboMethSeq [34,35] analyses, a similar trend for most of the residues is observed. For example, our analysis also demonstrated that ribose methylation at A100 catalyzed by snR51 in 18S rRNA is unambiguously hypomodified. Ribosome heterogeneity with respect to the rRNA modification is potentially one of the many pathways leading to specialized ribosomes as discussed recently [38]. The existence of specialized ribosomes implies likelihood of specialized translation that represent another layer of differential gene expression and like post-translational modifications of proteins may be important to respond to specific internal or external stresses, apart from playing vital roles in normal processes such as embryonic development and in diseases such as in cancer [39]. Nevertheless, this avenue of differential translation by virtue of ribosomal heterogeneity awaits further experimental demonstration. Although some of recent studies have provided supporting evidences in this direction [40,41]. Surprisingly, so far analyses in single cell organisms including yeast have not been able to decipher the precise role of these chemical modifications, although some modifications have been reported to affect translational speed and accuracy [31,42,43]. On the other hand, loss of these chemical modifications have been lethal in multicellular organisms, especially during early embryonic development [44]. Similarly, the machinery involved in rRNA modification has been linked to human diseases including cancer (listed in [10]), although precise contribution of rRNA modifications in the disease etiology remained to be established.

In summary, in the present study we provide a complete composition profile for ribose and base modifications of yeast rRNA. Each modification was precisely mapped with a single nucleotide resolution to its exact location on the ribosome. Additionally, we also calculated approximate molar amount for each modification that in turn reflect their stoichiometric levels. As observed recently by other techniques, we also confirmed sub-stoichiometric levels for some of the modified residues, emphasizing once again the ribosomal heterogeneity with respect to its repertoire of chemical modifications.

## Supporting Information

S1 Fig RP-HPLC chromatogram of a standard aqueous mixture of commercially available ribonucleosides. Identities of relevant peaks are mentioned in the table below.
(PDF)

S2 Fig Mapping of 2’O-methylguanosines (Gm) and 2’O-methylcytidines (Cm) of 18S rRNA using snoRNA deletion mutants. To corroborate the location of residues mapped by mung bean nuclease assay, snoRNA deletion mutants for the respective ribose methylations were used. A specific loss of peak corresponding to the respective modification in a deletion mutant validated the precise location of the modified residue. Overlaid RP-HPLC chromatograms of the MBN protected fragments containing (A) Gm562 isolated from wild type (WT) and ∆snr40 deletion mutant, (B) Gm1126 isolated from WT and ∆snr41 deletion mutant, (C) Gm1271 isolated from WT and ∆snr40 deletion mutant, (D) Gm1428 isolated from WT and ∆snr56 deletion mutant, (E) Gm1572 isolated from WT and ∆snr57 deletion mutant. To map and calculate the extent of each Cm modification in 18S rRNA, we deleted corresponding snoRNAs and calculated the contribution of individual peak to the total Cm peak area. F) Overlaid chromatograms of 18S rRNA derived nucleosides, isolated from isogenic WT, ∆snr70, and double mutant ∆snr70∆snr79. To validate the precise location of ac^4^C 1773, a rDNA point mutant was used where C1773 was exchanged with G in a plasmid-borne copy of 35S rDNA transcribed under the native promoter in a strain where the genomic rDNA was deleted. Exchange of C1773 to G led to 50% reduction in the amount of ac^4^C derived from 18S rRNA. (G) Overlaid chromatograms of isogenic WT and C1773G rDNA point mutant.
(PDF)

S3 Fig Mapping of base modifications 25S rRNA using rDNA point mutants. To validate the location of base modifications of 25S rRNA, rDNA point mutants were generated where the modified residues were point mutated in a plasmid-containing 35S rDNA transcribed under the control of native promoter in a strain with genomic rDNA deletion. Overlaid chromatograms of isogenic WT and A645U rDNA point mutant (A), isogenic WT and A2142U rDNA point mutant (B), isogenic WT and C2278G rDNA point mutant (C), isogenic WT and U2634G rDNA point mutant (D), and isogenic WT and U2843C rDNA point mutant (E). As for snoRNA deletion mutant specific loss of the modification in rDNA mutant validated the precise location of the corresponding modification.
(PDF)

S1 Table Yeast strains used in the present study. (PDF)

S2 Table Oligonucleotides used in the present study for MBN protection assay. (PDF)

S3 Table 18S rRNA fragments (isolated by MBN digestion) with their respective modifications profile. (PDF)

S4 Table 25S rRNA fragments (isolated by MBN digestion) with their respective modifications profile. (PDF)
