# Correction: Mapping of Complete Set of Ribose and Base Modifications of Yeast rRNA by RP-HPLC and Mung Bean Nuclease Assay

**DOI:** 10.1371/journal.pone.0173940

**Published:** 2017-03-09

**Authors:** Jun Yang, Sunny Sharma, Peter Watzinger, Johannes David Hartmann, Peter Kötter, Karl-Dieter Entian

In Table 1, there are missing column headers. Please see the complete, correct Table 1 here.

**Table 1:** RP-HPLC quantification of nucleosides in S.cerevisiae 18S and 25S rRNA.

	18S rRNA		25S rRNA	
*Nucleosides*	*Mole % (Seq)*	*Mole % (HPLC)*	*Mole % (Seq)*	*Mole %(HPLC)*
C (Cytidine)	19.1	19.5 ± 0.48	19.2	19.4 ± 0.56
U (Uridine)	27.4	27.1 ± 0.30	24.4	24.9 ± 0.39
G (Guanosine)	25.2	25.7 ± 0.12	28.2	27.5 ± 0.17
A (Adenosine)	26.3	26.6 ± 0.68	26.1	26.8 ± 0.64
	*Residues/mole*	*Residues/mole* *(HPLC)*	*Residues/mole*	*Residues/mole* *(HPLC)*
m^1^A (N1-Methyladenosine)	0	0	2	1.98 ± 0.10
m^5^C (C5-Methylcytidine)	0	0	2	1.81 ± 0.17
Cm (2’-O-Methylcytidine)	3	3.1 ± 0.26	7	6.74 ± 0.31
m^7^G (N7-Methylguanosine)	1	0.72*± 0.16	0	0
m^5^U (C5-Methyluridine)	0	0	0	0
Um (2’-O-Methyluridine)	2	1.89 ± 0.33	8	7.1 ± 0.31
m^3^U (N3-Methyluridine)	0	0	2	2.1 ± 0.17
Gm (2’-O-Methylguanosine)	5	4.91 ± 0.27	10	9.3 ± 0.39
ac^4^C (N4-Acetylcytidine)	2	2.12 ± 0.11	0	0
Am (2’-O-Methyladenosine)	8	7.33 ± 0.43	12	11.8 ± 0.48
m^6^_2_A (N6,N6-Dimethyladenosine)	2	nc	0	0

Mole % (Seq) was calculated from the number of each nucleoside in 18S and 25S rRNA sequences and as explained in Materials and Methods. Mole % (HPLC), Residues/mole (HPLC) and standard deviation values were calculated from the peak area of the respective nucleosides using their standard response factors, from nine independent runs for each 18S and 25S rRNA.

nc—not calculated

* m^7^G has been shown to undergo partial-degradation during hydrolysis [18].

There are several errors in Table 2. Please see the correct Table 2 here.

**Table 2:** Approximate values for the extent of modifications in 18S and 25S rRNA, using RP-HPLC and its comparison with RiboMethSeq and SILNAS analyses.

Modified residue	Made by	% of modification (RP-HPLC)	% of modification (Ribo-Meth Seq) (Ref.35)	% of modification (Ribo-Meth Seq) (Ref.34)	% of modification (SILNAS) (Ref.37)
**18S rRNA**					
**Am28**	snR74	90% ± 3.8%	85%	99%	>95%
**Am100**	snR51	73% ± 2.4%	77%	77%	80%
**Cm414**	U14	89% ± 3.1%	89%	96%	>95%
**Am420**	snR52	98% ± 1.5%	83%	90%	>95%
**Am436**	snR87	98% ± 1.8%	74%	89%	73%
**Am541**	snR41	95% ± 3.2%	87%	99%	>95%
**Gm562**	snrR40	100%	94%	92%	67%
**Um578**	snR77	95% ± 3.3%	90%	93%	>95%
**Am619**	snR47	98% ± 1.2%	85%	92%	100%
**Am796**	snR53	98% ± 1.1%	92%	97%	>95%
**Am974**	snR54	95% ± 3.4%	91%	96%	94%
**Cm1007**	snR79	100%	92%	98%	>95%
**Gm1126**	snR41	100%	94%	93%	89%
**m**^**1**^**acp**^**3**^**Ψ1191**	snR35-Nep1-Tsr3	nd	nd	nd	100%
**Um1269**	snR55	95% ± 2.1%	88%	92%	>95%
**Gm1271**	snR40	100%	92%	98%	>95%
**ac**^**4**^**C1280**	Kre33	96% ± 1.9%	nd	nd	84%
**Gm1428**	snR56	100%	95%	99%	>95%
**Gm1572**	snR57	100%	89%	97%	100%
**m**^**7**^**G1575**	Bud23	72*% ± 2.8%	nd	nd	>95%
**Cm1639**	snR70	92% ± 2.9%	93%	98%	79%
**ac**^**4**^**C1773**	Kre33	100%	nd	nd	>95%
**m**^**6**^_**2**_**A1781**	Dim1	nc	nd	nd	90%
**m**^**6**^_**2**_**A1782**	Dim1	nc	nd	nd	90%
**25S rRNA**					
**m**^**1**^**A645**	Rrp8	99% ± 1%	nd	nd	>95%
**Am649**	U18	93% ± 2.5%	88%	98%	>95%
**Cm650**	U18	100%	95%	98%	94%
**Cm663**	snR58	81% ± 4.3%	62%	91%	74%
**Gm805**	snR39b	90% ± 4.7%	93%	98%	>95%
**Am807**	snR39_snR59	98% ± 1%	94%	97%	>95%
**Am817**	snR60	98% ± 0.8%	92%	85%	89%
**Gm867**	snR50	90% ± 3.7%	95%	95%	78%
**Am876**	snR72	95% ± 2.2%	89%	91%	75%
**Um898**	snR40	94% ± 1.8%	78%	85%	94%
**Gm908**	snR60	95% ± 1.1%	96%	96%	100%
**Am1133**	snR61	98% ± 0.5%	92%	99%	88%
**Gm1142**	?(snr57)	absent	absent	absent	absent
**Cm1437**	U24	100%	92%	97%	>95%
**Am1449**	U24	97% ± 0.7%	67%	99%	>95%
**Gm1450**	U24	100%	94%	99%	>95%
**Um1888**	snR62	99% ± 1.3%	94%	97%	>95%
**m**^**1**^**A2142**	Bmt2	99% ± 0.7%	nd	nd	>95%
**Cm2197**	snR76	100%	71%	86%	91%
**Am2220**	snR47	98% ± 3.1%	94%	99%	93%
**Am2256**	snR63	98% ± 2.3%	94%	95%	100%
**m**^**5**^**C2278**	Rcm1	100%	nd	nd	100%
**Am2280**	snR13	99% ± 0.6%	91%	100%	100%
**Am2281**	snR13	99% ± 0.9%	92%	100%	100%
**Gm2288**	snR75	100%	95%	96%	>95%
**Cm2337**	snR64	100%	94%	99%	100%
**Um/Ψm 2347**	snR65/snR9	66% ± 3.7%	69%	61%	76.2%/12.9%
**Gm2395**	?snR190	absent	absent	absent	absent
**Um2417**	snR66	99% ± 0.1%	95%	99%	100%
**Um2421**	snR78	99% ± 1.5%	86%	93%	>95%
**Gm2619**	snR67	100%	93%	98%	87%
**m**^**3**^**U2634**	Bmt5	100%	nd	nd	>95%
**Am2640**	snR68	95% ± 3.6%	90%	96%	93%
**Um2724**	snR67	92% ± 2.1%	92%	98%	>95%
**Um2729**	snR51	89% ± 4.3%	67%	94%	75%
**Gm2791**	snR48	95% ± 2.3%	94%	98%	86%
**Gm2793**	snR48	95% ± 1.9%	95%	98%	>95%
**Gm2815**	snR38	95% ± 2%	95%	99%	>95%
**m**^**3**^**U2843**	Bmt6	100%	nd	nd	>95%
**m**^**5**^**C2870**	Nop2	100%	nd	nd	>95%
**Um2921**	snR52	93% ± 2.2%	92%	99%	>95%
**Gm2922**	Spb1	97% ± 3.5%	95%	98%	>95%
**Am2946**	snR71	99% ± 0.6%	92%	99%	91%
**Cm2948**	snR69	99% ± 0.8%	77%	82%	>95
**Cm2959**	snR73	93% ± 4.1%	96%	95%	94%

nd—not detected; nc—not calculated.? (snRx)- predicted according to the guide sequence; lacks experimental evidence

% modification was calculated as a mean of three independent calculations ± Relative standard deviations (RSD).

m^7^G has been shown to undergo partial-degradation during hydrolysis [18].
