# Supplementary material for: Mapping of Complete Set of Ribose and Base Modifications of Yeast rRNA by RP-HPLC and Mung Bean Nuclease Assay
Source: PLoS One. 2016 Dec 29;11(12):e0168873. doi: 10.1371/journal.pone.0168873 (PMC5199042; doi:10.1371/journal.pone.0168873)
Supplement: S1 Fig — Identities of relevant peaks are mentioned in the table below. (PDF) [file pone.0168873.s001.pdf]

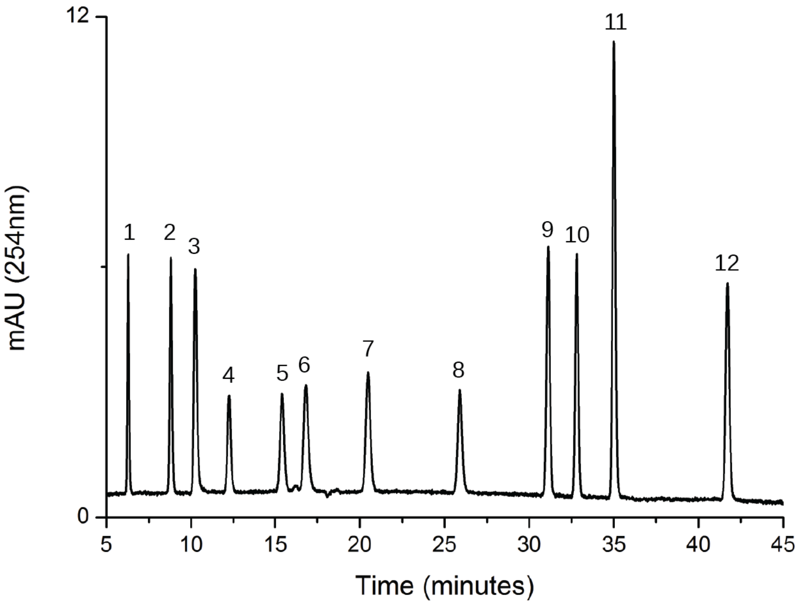

1	2	3	4	5	6	7	8	9	10	11	12
C	U	m^1A	m^5C	Cm	m^7G	G	m^3U	Gm	ac^4C	A	Am

S1 Fig. *RP-HPLC chromatogram of a standard aqueous mixture of commercially available ribonucleosides. Identities of relevant peaks are mentioned in the table below.*
