# Supplementary material for: Mapping of Complete Set of Ribose and Base Modifications of Yeast rRNA by RP-HPLC and Mung Bean Nuclease Assay
Source: PLoS One. 2016 Dec 29;11(12):e0168873. doi: 10.1371/journal.pone.0168873 (PMC5199042; doi:10.1371/journal.pone.0168873)
Supplement: S2 Fig — To corroborate the location of residues mapped by mung bean nuclease assay, snoRNA deletion mutants for the respective ribose methylations were used. A specific loss of peak corresponding to the respective modification in a deletion mutant validated the precise location of the modified residue. Overlaid RP-HPLC chromatograms of the MBN protected fragments containing (A) Gm562 isolated from wild type (WT) and ∆snr40 deletion mutant, (B) Gm1126 isolated from WT and ∆snr41 deletion mutant, (C) Gm1271 isolated from WT and ∆snr40 deletion mutant, (D) Gm1428 isolated from WT and ∆snr56 deletion mutant, (E) Gm1572 isolated from WT and ∆snr57 deletion mutant. To map and calculate the extent of each Cm modification in 18S rRNA, we deleted corresponding snoRNAs and calculated the contribution of individual peak to the total Cm peak area. F) Overlaid chromatograms of 18S rRNA derived nucleosides, isolated from isogenic WT, ∆snr70, and double mutant ∆snr70∆snr79. To validate the precise location of ac4C 1773, a rDNA point mutant was used where C1773 was exchanged with G in a plasmid-borne copy of 35S rDNA transcribed under the native promoter in a strain where the genomic rDNA was deleted. Exchange of C1773 to G led to 50% reduction in the amount of ac4C derived from 18S rRNA. (G) Overlaid chromatograms of isogenic WT and C1773G rDNA point mutant. (PDF) [file pone.0168873.s002.pdf]

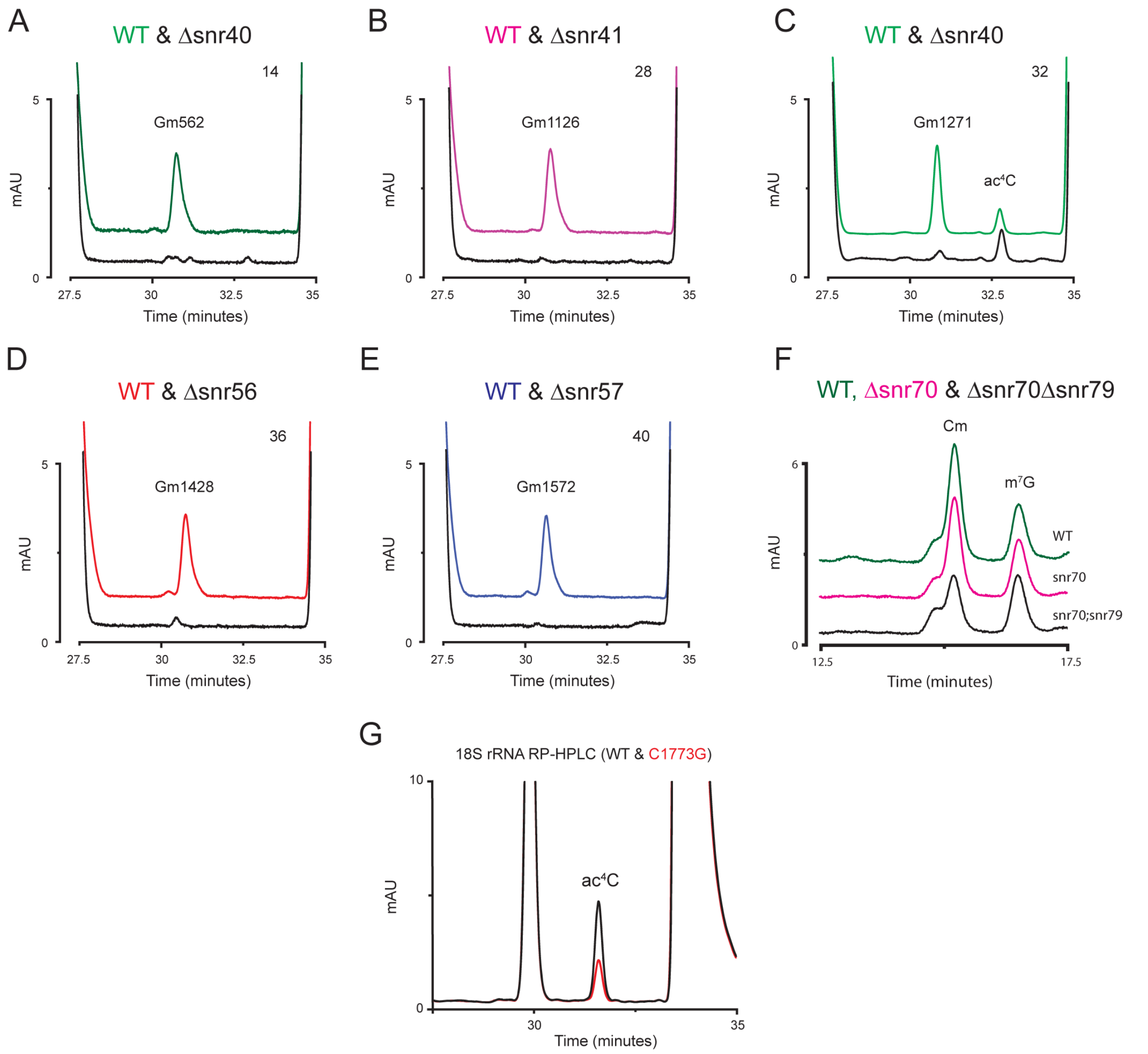

S2 Fig**. **Mapping of 2'O-methylguanosines (Gm) and 2'O-methylcytidines (Cm) of 18S rRNA using snoRNA deletion mutants. *To corroborate the location of residues mapped by mung bean nuclease assay, snoRNA deletion mutants for the respective ribose methylations were used. A specific loss of peak corresponding to the respective modification in a deletion mutant validated the precise location of the modified residue. Overlaid RP-HPLC chromatograms of the MBN protected fragments containing (A) Gm562 isolated from wild type (WT) and Δsnr40 deletion mutant, (B) Gm1126 isolated from WT and Δsnr41 deletion mutant, (C) Gm1271 isolated from WT and Δsnr40 deletion mutant, (D) Gm1428 isolated from WT and Δsnr56 deletion mutant, (E) Gm1572 isolated from WT and Δsnr57 deletion mutant. To map and calculate the extent of each Cm modification in 18S rRNA, we deleted corresponding snoRNAs and calculated the contribution of individual peak to the total Cm peak area. F) Overlaid chromatograms of 18S rRNA derived nucleosides, isolated from isogenic WT, Δsnr70, and double mutant Δsnr70Δsnr79. To validate the precise location of* ac^4C *1773, a rDNA point mutant was used where C1773 was exchanged with G in a plasmid-borne copy of 35S rDNA transcribed under the native promoter in a strain where the genomic rDNA was deleted. Exchange of C1773 to G led to 50% reduction in the amount of* ac^4C *derived from 18S rRNA. (G) Overlaid chromatograms of isogenic WT and C1773G rDNA point mutant.*
