# Supplementary material for: Mapping of Complete Set of Ribose and Base Modifications of Yeast rRNA by RP-HPLC and Mung Bean Nuclease Assay
Source: PLoS One. 2016 Dec 29;11(12):e0168873. doi: 10.1371/journal.pone.0168873 (PMC5199042; doi:10.1371/journal.pone.0168873)
Supplement: S3 Fig — To validate the location of base modifications of 25S rRNA, rDNA point mutants were generated where the modified residues were point mutated in a plasmid-containing 35S rDNA transcribed under the control of native promoter in a strain with genomic rDNA deletion. Overlaid chromatograms of isogenic WT and A645U rDNA point mutant (A), isogenic WT and A2142U rDNA point mutant (B), isogenic WT and C2278G rDNA point mutant (C), isogenic WT and U2634G rDNA point mutant (D), and isogenic WT and U2843C rDNA point mutant (E). As for snoRNA deletion mutant specific loss of the modification in rDNA mutant validated the precise location of the corresponding modification. (PDF) [file pone.0168873.s003.pdf]

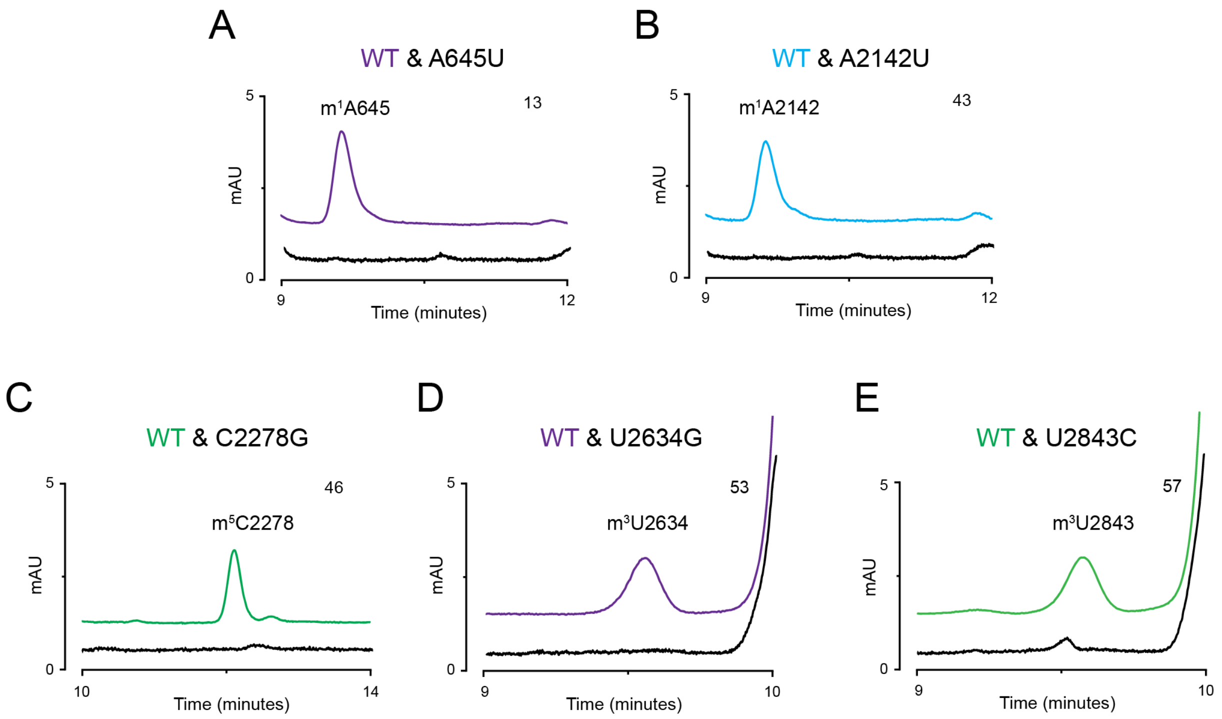

S3 Fig. Mapping of base modifications 25S rRNA using rDNA point mutants. *To validate the location of base modifications of 25S rRNA, rDNA point mutants were generated where the modified residues were point mutated in a plasmid-containing 35S rDNA transcribed under the control of native promoter in a strain with genomic rDNA deletion. Overlaid chromatograms of isogenic WT and A645U rDNA point mutant (A), isogenic WT and A2142U rDNA point mutant (B), isogenic WT and C2278G rDNA point mutant (C), isogenic WT and U2634G rDNA point mutant (D), and isogenic WT and U2843C rDNA point mutant (E). Like for snoRNA deletion mutant specific loss of the modification in rDNA mutant validated the precise location of the corresponding modification.*
