# Supplementary material for: Mapping of Complete Set of Ribose and Base Modifications of Yeast rRNA by RP-HPLC and Mung Bean Nuclease Assay
Source: PLoS One. 2016 Dec 29;11(12):e0168873. doi: 10.1371/journal.pone.0168873 (PMC5199042; doi:10.1371/journal.pone.0168873)
Supplement: S1 Table — (PDF) [file pone.0168873.s004.pdf]

S1 Table. Yeast strains used in the present study.

Strains	Alias	Genotypes	Source
CEN.PK1255-1C	C1773G	Mat a ura3-52 trp1-289 leu2-3, 112 his3Δ1 ΔΔrdn::pNOY455 [HIS3] +pPK760(1-1) C1773G G1788C	[1]
CEN.SO15-4B	Δsnr40	MATα ,ura3-52, his3-Δ1, leu2-3,112, trp1-289, MAL2-8c, SUC2, snr40::loxP-kanMX4::LEU2-loxP	[2]
CEN.SO20-1A	Δsnr41; Δsnr70, Δsnr51	MATa, ura3-52, his3-Δ1, leu2-3,112, trp1-289, MAL2-8c, SUC2, snr41-snr70-snr51::loxP-kanMX4::HIS3-loxP	[2]
CEN.SO29-8C	Δsnr56	MATα, ura3-52, his3-Δ1, leu2-3,112, trp1-289, MAL2-8c, SUC2, snr56::loxP-kanMX4::HIS3-loxP	[2]
CEN.SO32-4A	Δsnr57	MATa, ura3-52, his3-Δ1, leu2-3,112, trp1-289, MAL2-8c, SUC2, snr57::loxP-kanMX4::URA3-loxP	[2]
CEN.PK1170-1C	C2278G	MATa ura3-52 trp1-289 leu2-3,112 his3Δ1 ΔΔrDNA::pNOY455 [HIS3] + pPK655 (C2278G)	[3]
CEN.PK1168-1C	A2142T	MATa ura3-52 trp1-289 leu2-3,112 his3Δ1 ΔΔrdn::pNOY455 [HIS3] + pPK623 (A2142T)	[4]
CEN.PK1066-1C	A645T	MATa ura3-52 trp1-289 leu2-3,112 his3Δ1 MAL2-8^{C} SUC2 ΔΔrdn::pNOY455 [HIS3] + pPK618 (A645T)	[5]
CEN.JY11-1B	U2634G	MATa ura3-52 trp1-289 leu2-3,112 his3Δ1 ΔΔrdn::pNOY455 [HIS3] + pSJ05 (U2634G)	This study
CEN.JY12-4D	U2843C	MATa ura3-52 trp1-289 leu2-3,112 his3Δ1 MAL2-8^{C} SUC2 ΔΔrdn::pNOY455 + pSJ06 (U2843C)	This study
CEN.SH09	G1575A	MATa ura3-52 trp1-289 leu2-3,112 his3Δ1 MAL2-8^{C} SUC2 ΔΔrdn::pNOY455 [HIS3] + pSJ09 (G1575A)	This study
