# Supplementary material for: Mapping of Complete Set of Ribose and Base Modifications of Yeast rRNA by RP-HPLC and Mung Bean Nuclease Assay
Source: PLoS One. 2016 Dec 29;11(12):e0168873. doi: 10.1371/journal.pone.0168873 (PMC5199042; doi:10.1371/journal.pone.0168873)
Supplement: S2 Table — (PDF) [file pone.0168873.s005.pdf]

S2 Table. Oligonucleotides used in the present study for mung bean nuclease protection assay.

No.	Sequence (5'-->3')
18S-01	GGCTTAATCTTTGAGACAAGCATATGACTACTGGCAGGATCAACCAGATA
18S-02	GCCATTCGCAGTTTCACTGTATAAATTGCTTATACTTAGACATGCATGGC
18S-03	TAGTAAAGGAACTATCAAATAAACGATAACTGATTTAATGAGCCATTCGC
18S-04	AAGCATGTATTAGCTCTAGAATTACCACAGTTATACCATGTAGTAAAGGA
18S-05	TTATCTAATAAATACATCTCTTCCAAAGGGTCGAGATTTTAAGCATGTAT
18S-06	GTTATTATGAATCATCAAAGAGTCCGAAGACATTGATTTTTTATCTAATA
18S-07	GAATGAACCATCGCCAGCACAAGGCCATGCGATTCGAAAAGTTATTATGA
18S-08	CACTATCCTACCATCGAAAGTTGATAGGGCAGAAATTTGAATGAACCATC
18S-09	CGAACCCTTATTCCCCGTTACCCGTTGAAACCATGGTAGGCCACTATCCT
18S-10	CTTGGATGTGGTAGCCGTTTCTCAGGCTCCCTCTCCGGAATCGAACCCTT
18S-11	CTGAATTAGGATTGGGTAATTTGCGCGCCTGCTGCCTTCCTTGGATGTGG
18S-12	CCGAATGGGCCCTGTATCGTTATTTATTGTCACTACCTCCCTGAATTAGG
18S-13	GTTAAGGTATTTACATTGTACTCATTCCAATTACAAGACCCGAATGGGCC
18S-14	CGCGGCTGCTGGCACCAGACTTGCCCTCCAATTGTTCCTCGTTAAGGTAT
18S-15	CTGCAACAACTTTAATATACGCTATTGGAGCTGGAATTACCGCGGCTGCT
18S-16	CCGGCCAACCGGGCCCAAAGTTCAACTACGAGCTTTTTAACTGCAACAAC
18S-17	GAAAGGCCCCGTTGGAAATCCAGTACACGAAAAAATCGGACCGGCCAACC
18S-18	GGTTCGCCAAGAGCCACAAGGACTCAAGGTTAGCCAGAAGGAAAGGCCCC
18S-19	GCCTGCTTTGAACACTCTAATTTTTTCAAAGTAAAAGTCCTGGTTCGCCA
18S-20	TCCTATTCTATTATTCCATGCTAATATATTCGAGCAATACGCCTGCTTTG
18S-21	CATTACGATGGTCCTAGAAACCAACAAAATAGAACCAAACGTCCTATTCT
18S-22	CAATTGAATACTGATGCCCCCGACCGTCCCTATTAATCATTACGATGGTC
18S-23	GTAGTTAGTCTTCAATAAATCCAAGAATTTCACCTCTGACAATTGAATAC
18S-24	TTCTTGATTAATGAAAACGTCCTTGGCAAATGCTTTCGCAGTAGTTAGTC
18S-25	CTACGACGGTATCTGATCATCTTCGATCCCCTAACTTTCGTTCTTGATTA
18S-26	CACCACCCGATCCCTAGTCGGCATAGTTTATGGTTAAGACTACGACGGTA
18S-27	CTTTGATTTCTCGTAAGGTGCCGAGTGGGTCATTAAAAAAACACCACCCG
18S-28	GTTTCAGCCTTGCGACCATACTCCCCCCAGAACCCAAAGACTTTGATTTC
18S-29	AGGCTCCACTCCTGGTGGTGCCCTTCCGTCAATTCCTTTAAGTTTCAGCC
18S-30	GACCTGGTGAGTTTCCCCGTGTTGAGTCAAATTAAGCCGCAGGCTCCACT
18S-31	CAAGAAAGAGCTCTCAATCTGTCAATCCTTATTGTGTCTGGACCTGGTGA
18S-32	CACCAACTAAGAACGGCCATGCACCACCACCCACAAAATCAAGAAAGAGC
18S-33	GGTCTCGTTCGTTATCGCAATTAAGCAGACAAATCACTCCACCAACTAAG
18S-34	GATAACCAGCAAATGCTAGCACCACTATTTAGTAGGTTAAGGTCTCGTTC
18S-35	CTTCCATCGGCTTGAAACCGATAGTCCCTCTAAGAAGTGGATAACCAGCA
18S-36	GAACGTCTAAGGGCATCACAGACCTGTTATTGCCTCAAACTTCCATCGGC
18S-37	GACTCGCTGGCTCCGTCAGTGTAGCGCGCGTGCGGCCCAGAACGTCTAAG
18S-38	GGAGTTTCACAAGATTACCAAGACCTCTCGGCCAAGGTTAGACTCGCTGG
18S-39	TTGAAGAGCAATAATTACAATGCTCTATCCCCAGCACGACGGAGTTTCAC
18S-40	CAACGCAAGCTGATGACTTGCGCTTACTAGGAATTCCTCGTTGAAGAGCA
18S-41	ACTAGCGACGGGCGGTGTGTACAAAGGGCAGGGACGTAATCAACGCAAGC
18S-42	CTAAGCAGATCCTGAGGCCTCACTAAGCCATTCAATCGGTACTAGCGACG
18S-43	GTCCAAATTCTCCGCTCTGAGATGGAGTTGCCCCCTTCTCTAAGCAGATC
18S-44	CTTGTTACGACTTTTAGTTCCTCTAAATGACCAAGTTTGTCCAAATTCTC
18S-45	TAATGATCCTTCCGCAGGTTCACCTACGGAAACCTTGTTACGACTTTTAG
25S-01	CCTCCGCTTATTGATATGCTTAAGTTCAGCGGGTACTCCTACCTGATTTGAGGTCAAAC

25S-02	GCTTTTGCCGCTTCACTCGCCGTTACTAAGGCAATCCCGGTTGGTTTCTTTTCCTCCGC
25S-03	CCCTCTCCAAATTACAACTCGGGCACCGAAGGTACCAGATTTCAAATTTGAGCTTTTGCC
25S-04	CTCTATGACGTCCTGTTCCAAGGAACATAGACAAGGAACGGCCCCAAAGTTGCCCTCTCC
25S-05	CGAAGGCACTTTACAAAGAACCGCACTCCTCGCCACACGGGATTCTCACCCTCTATGACG
25S-06	GATGGAATTTACCACCCACTTAGAGCTGCATTCCCAAACAACTCGACTCTTCGAAGGCAC
25S-07	CATCACTGTACTTGTTCGCTATCGGTCTCTCGCCAATATTTAGCTTTAGATGGAATTTAC
25S-08	CAATTTCACGTACTTTTTCACTCTCTTTTCAAAGTTCTTTTCATCTTTCCATCACTGTAC
25S-09	GAGCAGAGGGCACAAAACACCATGTCTGATCAAATGCCCTTCCCTTTCAACAATTTCACG
25S-10	CACCAAAACTGATGCTGGCCCAGTGAAATGCGAGATTCCCCTACCCACAAGGAGCAGAGG
25S-11	GGCTATAATACTTACCGAGGCAAGCTACATTCCTATGGATTTATCCTGCCACCAAAACTG
25S-12	CCTTGACTTACGTCGCAGTCCTCAGTCCCAGCTGGCAGTATTCCCACAGGCTATAATAC
25S-13	CCTTGGTCCGTGTTTCAAGACGGGCGGCATATAACCATTATGCCAGCATCCTTGACTTAC
25S-14	CTTTCATTACGCGTATGGGTTTTACACCCAAACACTCGCATAGACGTTAGACTCCTTGGT
25S-15	CATCAGGATCGGTCGATTGTGCACCTCTTGCGAGGCCCCAACCTACGTTCACTTTCATTAC
25S-16	CCATCTTTCGGGTCCCAACAGCTATGCTCTTACTCAAATCCATCCGAAGACATCAGGATC
25S-17	GAGCCTCCACCAGAGTTTCCTCTGGCTTCACCCTATTCAGGCATAGTTCACCATCTTTCG
25S-18	CGCCCCTATACCCAAATTCGACGATCGATTTGCACGTCAGAACCGCTACGAGCCTCCACC
25S-19	GAGGGAAACTTCGGCAGGAACCAGCTACTAGATGGTTCGATTAGTCTTTCGCCCCTATAC
25S-20	CTAATCATTCGCTTTACCTCATAAAACTGATACGAGCTTCTGCTATCCTGAGGGAAACTTC
25S-21	CATATTTAAAGTTTGAGAATAGGTCAAGGTCATTTCGACCCCGGAACCTCTAATCATTCG
25S-22	GCTCTTCATTCAAATGTCCACGTTCAATTAAGTAACAAGGACTTCTTACATATTTAAAG
25S-23	CGGTTCATCCCGCATCGCCAGTTCTGCTTACCAAAAATGGCCCACTAAAAGCTCTTCATTC
25S-24	CCTTTTGTGGTGTCTGATGAGCGTGTATTCCGGCACCTTAACTCTACGTTCGGTTCATCC
25S-25	GCGGATTCCGACTTCCATGGCCACCGTCCGGCTGTCTAGATGAACTAACACCTTTTGTGG
25S-26	CCATTTTCAGGGCTAGTTCATTCGGCCGGTGAGTTGTTACACACTCCTTAGCGGATTCCG
25S-27	CATCATATCAACCCTGACGGTAGAGTATAGGTAACACGCTTGAGCGCCATCCATTTTCAG
25S-28	CTTACGGTCTAGGCTTCGTCACTGACCTCCACGCCTGCCTACTCGTCAGGGCATCATATC
25S-29	GAATATTTGCTACTACCACCAAGATCTGCACTAGAGGCCGTTCGACCCGACCTTACGGTC
25S-30	CTGCTGTTGACGTGGAACCTTTCCCCACTTCAGTCTTCAAAGTTCTCATTTGAATATTTGC
25S-31	CTTTGAAACGGAGCTTCCCCATCTCTTAGGATCGACTAACCCACGTCCAACTGCTGTTGAC
25S-32	CGGAATCTTAACCGGATTCCCTTTCGATGGTGGCCTGCATAAAATCAGGCCTTTGAAACG
25S-33	CGTCTCCACATTCAGTTACGTTACCGTGAAGAATCCATATCCAGGTTCCGGAATCTTAAC
25S-34	GGTGATAAGCTGTTAAGAAGAAAAGATAACTCCTCCCAGGGCTCGCGCCGACGTCTCCAC
25S-35	GTGCTGGCCTCTTCCAGCCATAAGACCCCATCTCCGGATAAACCAATTCCGGGGTGATAAG
25S-36	CTTCCTGTGGATTTTCACGGGCCGTCACAAGCGCACCGGAGCCAGCAAAGGTGCTGGCCTC
25S-37	CCTTGGAGACCTGCTGCGGTTATCAGTACGACCTGGCATGAAAACTATTCCTTCCTGTGG
25S-38	GCCGACTTCCCTTATCTACATTATTCTATCAACTAGAGGCTGTTCACCTTGGAGACC
25S-39	CTACCCGACCCTTAGAGCCAATCCTTATCCCGAAGTTACGGATCTATTTTGCCGACTTCC
25S-40	CCCACCAAGCAGTCCACAAGCACGCCCGCTGCGTCTGACCAAGGCCCTCACTACCCGACC
25S-41	CAAGGCCGTCTACAACAAGGCACGCAAGTAGTCCGCCTAGCAGAGCAAGCCCCACCAAGC
25S-42	GTTCTAAGTTGATCGTTAATTGTAGCAAGCGACGGTCTACAAGAGACCTACCAAGGCCGTC
25S-43	CGCAATGCTATGTTTTAATTAGACAGTCAGATTCCCCTTGTCCGTACCAGTTCTAAGTTG
25S-44	CAGAGCACTGGGCAGAAATCACATTGCGTCAACATCACTTTCTGACCATCGCAATGCTATG
25S-45	GTTACTCCCGCCGTTTACCCGCGCTTGGTTGAATTTCTTCACTTTGACATTCAGAGCACTG
25S-46	GCGCGTCACTAATTAGATGACGAGGCATTTGGCTACCTTAAGAGAGTCATAGTTACTCCC
25S-47	GTTTCGCTAGATAGTAGATAGGGACAGTGGGAATCTCGTTAATCCATTCATGCGCGTCAC
25S-48	CAGGGTCTTCTTTCCCCGCTGATTCTGCCAAGCCCGTTCCCTTGGCTGTGGTTTCGCTAG
25S-49	GAAGACCCTGTTGAGCTTGACTCTAGTTTGACATTGTGAAGAGACATAGAGGGTGTAG

25S-50	GAAACTATAAAGGTAGTGGTATTTCACTGGCGCCGAAGCTCCCACTTATTCTACACCCTC
25S-51	CTAGAACGTGGAAAATGAATTCCAGCTCCGCTTCATTGAATAAGTAAAGAAACTATAAAG
25S-52	CCACCTGACAATGTCTTCAACCCGGATCAGCCCCGAATGGGACCTTGAATGCTAGAACGTG
25S-53	CTTAGGACATCTGCGTTATCGTTTAACAGATGTGCCGCCCCAGCCAAACTCCCCACCTGAC
25S-54	GCTTTTACCCTTTTGTTCTACTGGAGATTTCTGTTCTCCATGAGCCCCCCTTAGGACATC
25S-55	GGCCACACTTTCATGGTTTGTATTCACACTGAAAATCAAAATCAAGGGGGCTTTTACCC
25S-56	GTAACTTTTCTGGCACCTCTAGCCTCAAATTCCGAGGGACTAAAGGATCGATAGGCCACAC
25S-57	GCAATGTCGCTATGAACGCTTGACTGCCACAAGCCAGTTATCCCTGTGGTAACTTTTCTG
25S-58	CGAATTCTGCTTCGGTATGATAGGAAGAGCCGACATCGAAGAATCAAAAAGCAATGTCGC
25S-59	CCCAGCTCACGTTCCCTATTAGTGGGTGAACAATCCAACGCTTACCGAATTCTGCTTCGG
25S-60	GGTAACATTCATCAGTAGGGTAAAACTAACCTGTCTCACGACGGTCTAAACCCAGCTCAC
25S-61	CAATTATCCGAATGAACTGTTCCTCTCGTACTAAGTTCAATTACTATTGCGGTAACATTC
25S-62	CCAGCGGATGGTAGCTTCGCGGCAATGCCTGATCAGACAGCCGCAAAAACCAATTATCCG
25S-63	GAAATCACCGCGTTCTAGCATGGATTCTGACTTAGAGGCGTTCAGCCATAATCCAGCGGATG
25S-64	GACGCCACAAGGACGCCTTATTCGTATCCATCTATATTGTGTGGAGCAAAGAAATCACCG
25S-65	CCCAAGGCCTTTCCGCCAAGTGCACCGTTGCTAGCCTGCTATGGTTCAGCGACGCCACAAG
25S-66	CAAATGATTTATCCCCACGCAAAATGACATTGCAATTCGCCAGCAAGCACCCAAGGCC
25S-67	CAAGGCTACTCTACTGCTTACAATACCCCGTTGTACATCTAAGTCGTATACAAATG
25S-68	ACAAATCAGACAACAAAGGCTTAATCTCAGCAGATCGTAACAACAAGGCTACTCTACTGC
18S-561	CAGACTTGCCCTCCAATTGTTCCTCGTTAAGGTATTTACATTGTACTCAT
18S-562	ACAACTTTAATATACGCTATTGGAGCTGGAATTACCGCGGCTGCTGGCAC
25S-2791	CTAGCCTCAAATTCCGAGGGACTAAAGGATCGATAGGCCACACTTTCATG
25S-2793	GAACGCTTGACTGCCACAAGCCAGTTATCCCTGTGGTAACTTTTCTGGCACC
25S-2265	CATTCATGCGCGTCACTAATTAGATGACGAGGCATTTGGCTACCTTAAGAG
18S-11a	CCTGCTGCCTTCCTTGGATGTGGTAGCCGTTTCTCAGGCTCCCTCTCCGG
18S-11b	CGTTATTTATTGTCACTACCTCCCTGAATTAGGATTGGGTAATTTGCGCGC
25S-60a	CGGTAACATTCATCAGTAGGGTAAAACTAACCTGTCTCACGACGGTCTAAAC
Oligo- 491	TCCTCGTTAAGGTATTTACATTGTACTCATTCCAATTACAAGACCCGAATG
Oligo-542	GAGCTGGAATTACCGCGGCTGCTGGCACCAGACTTGCCCTCCAATTGT
