# Supplementary material for: Mapping of Complete Set of Ribose and Base Modifications of Yeast rRNA by RP-HPLC and Mung Bean Nuclease Assay
Source: PLoS One. 2016 Dec 29;11(12):e0168873. doi: 10.1371/journal.pone.0168873 (PMC5199042; doi:10.1371/journal.pone.0168873)
Supplement: S3 Table — (PDF) [file pone.0168873.s006.pdf]

S3 Table. 18S rRNA fragments (isolated by Mung bean nuclease digestion) with their respective modifications profile.

No.	Ψ	Am	Cm	Gm	Um
18S-01		1			
18S-02					
18S-03	2	1			
18S-04					
18S-05	1				
18S-06	1				
18S-07	1				
18S-08					
18S-09					
18S-10			1		
18S-11		2	1		
18S-12	1				
18S-13					
18S-14		1		1	
18S-15					1
18S-16	1	1			
18S-17					
18S-18					
18S-19	2				
18S-20		1			
18S-21					
18S-22					
18S-23					

No.	Ψ	Am	Cm	Gm	Um	ac^4C	m^7G	m^6_2A	acp
18S-24		1							
18S-25	1	1	1						
18S-26			1						
18S-27									
18S-28				1					
18S-29				1					
18S-30	2								1
18S-31									
18S-32	1			1	1	1			
18S-33	1								
18S-34									
18S-35									
18S-36	1			1					
18S-37									
18S-38									
18S-39									
18S-40				1			1		
18S-41			1						
18S-42									
18S-43									
18S-44									
18S-45						1		2	
