# Supplementary material for: Mapping of Complete Set of Ribose and Base Modifications of Yeast rRNA by RP-HPLC and Mung Bean Nuclease Assay
Source: PLoS One. 2016 Dec 29;11(12):e0168873. doi: 10.1371/journal.pone.0168873 (PMC5199042; doi:10.1371/journal.pone.0168873)
Supplement: S4 Table — (PDF) [file pone.0168873.s007.pdf]

***S4 Table. 25S rRNA fragments (isolated by Mung bean nuclease digestion) with their respective modifications profile.***

| No. | Ψ | Am | Cm | Gm | Um | $m^1A$ |
|---|---|---|---|---|---|---|
| 25S-01 | | | | | | |
| 25S-02 | | | | | | |
| 25S-03 | | | | | | |
| 25S-04 | | | | | | |
| 25S-05 | | | | | | |
| 25S-06 | | | | | | |
| 25S-07 | | | | | | |
| 25S-08 | | | | | | |
| 25S-09 | | | | | | |
| 25S-10 | | | | | | |
| 25S-11 | | | | | | |
| 25S-12 | | | | | | |
| 25S-13 | | 1 | 1 | | | 1 |
| 25S-14 | | | 1 | | | |
| 25S-15 | | | | | | |
| 25S-16 | 1 | 1 | | 1 | | |
| 25S-17 | | 1 | | | | |
| 25S-18 | | 1 | | 2 | 1 | |
| 25S-19 | 1 | | | | | |
| 25S-20 | 5 | | | | | |
| 25S-21 | 4 | | | | | |
| 25S-22 | 2 | | | | | |
| 25S-23 | 2 | 1 | | 1 | | |
| 25S-24 | | | | | | |
| 25S-25 | | | | | | |
| 25S-26 | | | | | | |
| 25S-27 | | | | | | |
| 25S-28 | | | | | | |
| 25S-29 | | 1 | 1 | 1 | | |
| 25S-30 | | | | 1 | | |
| 25S-31 | | | | | | |
| 25S-32 | | | | | | |
| 25S-33 | | | | | | |
| 25S-34 | | | | | | |

| No. | Ψ | Am | Cm | Gm | Um | $m^1A$ | $m^3U$ | $m^5C$ |
|---|---|---|---|---|---|---|---|---|
| 25S-35 | | | | | | | | |
| 25S-36 | | | | | | | | |
| 25S-37 | | | | | | | | |
| 25S-38 | | | | | 1 | | | |
| 25S-39 | | | | | | | | |
| 25S-40 | | | | | | | | |
| 25S-41 | | | | | | | | |
| 25S-42 | | | | | | | | |
| 25S-43 | 2 | | | | | 1 | | |
| 25S-44 | 1 | | 1 | | | | | |
| 25S-45 | | 2 | 1 | | | | | |
| 25S-46 | 4 | 3 | | 1 | | | | 1 |
| 25S-47 | 4 | | 1 | | 1 | | | |
| 25S-48 | 1 | | | | | | | |
| 25S-49 | 1 | | | | 2 | | | |
| 25S-50 | | | | | | | | |
| 25S-51 | | | | | | | | |
| 25S-52 | | | | | | | | |
| 25S-53 | | 1 | | 1 | | | 1 | |
| 25S-54 | | | | | | | | |
| 25S-55 | 1 | | | | 2 | | | |
| 25S-56 | | | | 2 | | | | |
| 25S-57 | 1 | | | 1 | | | 1 | |
| 25S-58 | 1 | | | | | | | 1 |
| 25S-59 | 2 | 1 | 1 | 1 | 1 | | | |
| 25S-60 | 2 | 1 | 2 | | | | | |
| 25S-61 | | | | | | | | |
| 25S-62 | | | | | | | | |
| 25S-63 | | | | | | | | |
| 25S-64 | | | | | | | | |
| 25S-65 | | | | | | | | |
| 25S-66 | | | | | | | | |
| 25S-67 | | | | | | | | |
| 25S-68 | | | | | | | | |
